# At–Sea Behavior Varies with Lunar Phase in a Nocturnal Pelagic Seabird, the Swallow-Tailed Gull

**DOI:** 10.1371/journal.pone.0056889

**Published:** 2013-02-26

**Authors:** Sebastian M. Cruz, Mevin Hooten, Kathryn P. Huyvaert, Carolina B. Proaño, David J. Anderson, Vsevolod Afanasyev, Martin Wikelski

**Affiliations:** 1 Department of Migration and Immuno-Ecology, Max Planck Institute for Ornithology, Radolfzell, Baden-Württemberg, Germany; 2 U. S. Geological Survey, Colorado Cooperative Fish and Wildlife Research Unit, Fort Collins, Colorado, United States of America; 3 Department of Fish, Wildlife, and Conservation Biology, Colorado State University, Fort Collins, Colorado, United States of America; 4 Department of Biology, Wake Forest University, Winston-Salem, North Carolina, United States of America; 5 British Antarctic Survey, Natural Environment Research Council, Cambridge, United Kingdom; 6 Konstanz University, Konstanz, Baden-Württemberg, Germany; Institut Pluridisciplinaire Hubert Curien, France

## Abstract

Strong and predictable environmental variability can reward flexible behaviors among animals. We used long-term records of activity data that cover several lunar cycles to investigate whether behavior at-sea of swallow-tailed gulls *Creagrus furcatus*, a nocturnal pelagic seabird, varied with lunar phase in the Galápagos Islands. A Bayesian hierarchical model showed that nighttime at-sea activity of 37 breeding swallow-tailed gulls was clearly associated with changes in moon phase. Proportion of nighttime spent on water was highest during darker periods of the lunar cycle, coinciding with the cycle of the diel vertical migration (DVM) that brings prey to the sea surface at night. Our data show that at-sea behavior of a tropical seabird can vary with environmental changes, including lunar phase.

## Introduction

The lunar cycle influences the ecology, movements, and foraging behavior of many nocturnal organisms through its effect on light availability [1]–[3]. Predators such as owls, bats, and nightjars concentrate their activity within certain periods of the night and of the lunar cycle to maximize hunting success [4]. In contrast, some nocturnally active prey animals like rats, insects, and frogs alter their activity across the lunar cycle to avoid visual predators [5]–[7]. In marine systems, the lunar phase is known to influence the diel vertical migration (DVM) of zooplankton, squid, and fish, potential prey that feed at the ocean surface at night and hide at depth from visual surface predators during the day [8]–[10]. The extent of the DVM varies in concert with the degree of moonlight during the lunar cycle: the DVM is reduced on the brightest nights and is most extensive when the moon is in the new phase [11]–[13]. During dark nights, surface densities of prey can be a thousand times greater than during the day; this migration is more pronounced at low than high latitudes, and in pelagic than neritic waters [14].

The migration by prey in the DVM isolates them from most pelagic birds, many of which forage mostly or strictly during daylight [15]. Nonetheless, some marine predators can use celestial illumination effectively to obtain prey at night, especially as the lunar phase approaches full [16]. Common murres, *Uria aalge,* dive deeper under high nocturnal illumination, matching the DVM patterns of capelin, *Mallotus villosus*, their main prey [17]. Some species of albatross show a positive correlation between nocturnal flight activity and moon phase, and nighttime activity of petrels and shearwaters matches lunar phase: they fly more and land on water more frequently during full moon conditions, suggesting that nighttime visual foraging is more effective when ambient light level is highest [18]–[22].

These and other studies on the effects of the lunar cycle on seabird behavior involve species that are largely diurnal, but engage in some nighttime foraging activity [18], [21], [23]. A concern is that marine predators that rely on optical cues to forage effectively are constrained by their visual adaptations to hunt only in a specific light range [17], [24]. The swallow-tailed gull *Creagrus furcatus* is an oceanic nocturnal specialist that eats squid and small fish that rise to the surface at night, capturing them by surface plunging [15], [25]–[29] and Cruz et. al. unpublished data]. The diet of swallow-tailed gulls consists mainly of squid *Sthenoteuthis oualaniensis*, an abundant, vertically migrating species in the tropical Pacific, and also clupeid fish whose distribution varies vertically with time of day [28], [30]. The adaptations for nocturnal foraging in swallow-tailed gulls include large eyes with a layer of tissue, the *tapetum lucidum*, that reflects visible light back through the retina, increasing the light available to the eye’s photoreceptors [29]. Similar traits have evolved in a range of nocturnal predators and are thought to increase foraging efficiency [24], [31]. Given these adaptations, the swallow-tailed gull may not be subject to the visual constraints imposed by darkness that affect many other species of seabird, such that this species presents an excellent opportunity to study the relationships between the phases of the moon and at-sea behavior in a nocturnal specialist.

In this paper we examine activity data that cover several consecutive lunar cycles to explore whether at-sea behavior varies with lunar phase in swallow-tailed gulls, complementing the extensive existing work on diurnal species [17], [19], [20], [22], [32]. Recently developed global location sensors (GLS) equipped with wet/dry loggers can record bird activity over long periods of time [33]. Recent studies have used this technology successfully to investigate a wide range of questions regarding seabird activity at-sea [17], [21], [22]. We deployed GLS units on a sample (n = 50) of swallow-tailed gulls, to explore their at-sea behavior in relation to lunar phase.

We tested the hypothesis that swallow-tailed gulls maximize their foraging activity when prey is most available. Accordingly, we predicted that foraging activity, measured as the proportion of nighttime spent on water, is higher during darker periods of the lunar cycle, coinciding with the strongest DVM and highest prey density. We included sea surface temperature (SST) as a factor in our analysis because a mild El Niño Southern Oscillation (ENSO) event occurred during the study period, which increased the variability of SST (Climate Prediction Center, National Centers for Environmental Prediction NOAA/National Weather Service). Large fluctuations in the amplitude of SST during ENSO events influence seabird reproduction, probably mediated by temperature-related changes in the abundance of marine prey [34], [35].

## Materials and Methods

### Study Site and Sensor Deployment and Recovery

Archival global location sensors (GLS) with a wet/dry sensor (MK14, mass 1.5 g; size 20×9×5.5 mm; British Antarctic Survey) were deployed on 50 adult swallow-tailed gulls between 18–21 October, 2009 at Punta Cevallos, Española Island, Galápagos, Ecuador (1° 23′S, 89°37′W). Swallow-tailed gulls breed asynchronously; this study included adults at the egg laying (n = 33), incubating (n = 9), chick rearing (n = 1), and fledgling (n = 7) stages. Birds were captured by hand at their nest or while resting on rocks and then held by one member of a two-person team. Each bird was fitted on its left leg with a plastic band (Pro-Touch Engraving, Canada) to which the GLS had been attached earlier with epoxy resin and cable ties (mass of logger, band, epoxy and cable tie: 2 g, ∼0.3% of adult body mass). Bird capture and handling times were ∼5 min during logger deployment and recovery. We did not detect any adverse effects from handling or tag attachment on reproduction as none of the tagged breeding birds abandoned their nests or other parental care in the days following tag deployment. Recaptures occurred at different times during 2010 and 2011 due to the asynchronous breeding schedules of swallow-tailed gulls, such that the deployment period for each bird was between six and sixteen months. Each logger was equipped with a wet/dry sensor that detects immersion in seawater. Wet or dry status was recorded every 3 s as a 1 or 0; these data were summed over 10 min intervals by the loggers, providing a value from 0 to 200 that represents the proportion of time an individual spent in the water during each 10 min period. The Galápagos National Park Service approved of and granted the research permits for this work.

### Post-deployment Data Processing and Analysis

Immersion data, our indicator of foraging activity, were uploaded and decompressed with BAStrak software version 8 (British Antarctic Survey, March 2010). The raw data from the unit were values from 0 to 200, indicating the number of 3 s periods during 10 min blocks that the sensor on the unit was wet. We were interested in the proportion of time that breeding individuals spent in the water at night. To calculate this, the values from all 10 min blocks (blocks per night = 72) were summed for each night, providing an aggregated count of 3 s sub-periods in which a bird was on the water. These were transformed to the total proportion of time a bird was in the water by finding the quotient of the aggregate count and 14,400, the maximum count possible (72 * 200 = 14,400). Nighttime was defined as the 12-hour period between 18∶00 and 6∶00 local Galápagos time, appropriate for this equatorial location.

To examine activity patterns in further detail we calculated the number of wet bouts per night during the breeding season. Wet bouts were not used in our model they are only presented graphically in our results. A wet bout was defined as a continuous sequence of 10 min blocks during each of which the bird spent at least 3 s on the water. Alternatively, during a flying bout every 10 min block was completely dry. Night-time wet bouts were estimated using our immersion data following Phalan et al [19].

An ENSO episode was underway in 2010 during a portion of our study period (Climate Prediction Center, National Centers for Environmental Prediction NOAA/National Weather Service). Because ENSO conditions are known to affect seabirds [35] we included this as a temporal covariate in our analysis of swallow-tailed gull at-sea behavior.

Data for SST were obtained from the Charles Darwin Foundation Climate Database (http://www.darwinfoundation.org/datazone/climate/). The fraction of the moon illuminated each night was obtained using calendars from the U.S Naval Observatory and Astronomical Applications Department (http://aa.usno.navy.mil/data/docs/MoonFraction.php).

We used R 2.13.1 (The R Foundation for Statistical Computing, Vienna, Austria) for data management and statistical analyses and the R package ggplot2 (H. Wickham, New York, 2009) for graphics.

### Model Specification

We monitored *m* total birds over a sequence of *T* total nightly periods. For these observations, we recorded a discrete response variable for each gull, because we were interested primarily in the effect of the lunar phase on at-sea activity, and the sensor was able to detect wet versus dry as a binary response, such that the variable we actually modeled is a count, *y_i,t_* that is less than or equal to 14,400 (the maximum number of wet 3 second sub-periods in 12 hours). In order to model these counts such that we could quantify the probability of wet versus dry on each 12-hour nightly period, we assumed a binomial model for the 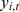 with:
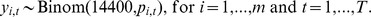



To complete the model specification so that we could make inference on a set of potentially influential covariates, the wet probabilities, 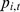
were linked formally to the environmental conditions. The traditional way to accomplish this is to express the logit link function of the 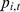 as a linear combination of the effects. That is, consider the generalized linear mixed model (GLMM) specification:
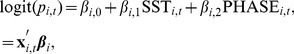
(1)where the 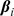 coefficients correspond to the potential effects specific to bird 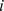, and *i* are the covariates relative to bird *t* on nightly period *t*. In this case, the covariates were the sea surface temperature and moon phase (ranging from zero for new to one for full).In this model, each bird can have its own reaction to the environment (including moon phase), in terms of their behavior at-sea, but we were ultimately interested in the population-level response to moon phase. The model in (1) uses the nightly 12-hour period as the sample unit, yet we wanted to draw inferences about the effects of the covariates using individual birds as the sample units to allow population-level inference. We assumed that each coefficient vector 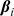 came from a population-level distribution (as a random effect). We desired inference on the mean of this distribution 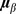
where:



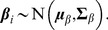
(2)The latent process model presented in (2) implies a hierarchical specification for the GLMM and we had two components that needed prior distributions. In completing the model specification, 
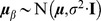
 let and where, 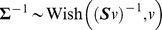, the prior for the inverse covariance matrix 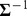 is a Wishart distribution, a proper probability distribution for precision matrices. This model allows the 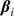 coefficient vectors to be correlated.

We used a Markov Chain Monte Carlo (MCMC) algorithm to sample from the posterior distribution of the unknown parameters given the data. In this case, the posterior distribution can be written as proportional to the likelihood multiplied by the process model and prior as follows:
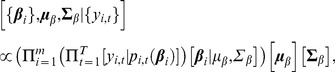
(3)where the square bracket notation [·|·] corresponds to a probability density function.

By sampling from each of the full-conditional distributions sequentially, one can implement an MCMC and obtain samples from the joint posterior distribution of interest [36]. In this model, we used hyper parameters 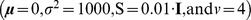 and ran the MCMC algorithm for 10,000 iterations, discarding the first 1,000 iterations as a burn-in period (i.e., the period before the Markov chains have converged).

An advantage of this hierarchical model specification was that we could account directly for the uncertainty present in the original data while allowing rigorous inference to be made on the population-level effects 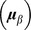.

## Results

Forty-six of 50 devices (92%) were recovered, and data were successfully uploaded from 45 (98%). Thirty-seven of the 45 (82%) loggers recorded at least one breeding attempt per individual, which average 120 days [28]. Breeding attempts were considered to occur between migration events; apparent breeding periods before the first migration and after the last migration were not included. We analyzed movement data derived from the GLS loggers to determine migration periods, with migration defined as a period of 3 moths or more when birds are away from the Galapagos Islands. Swallow-tailed gulls breed asynchronously, and the breeding periods of different birds overlapped to varying degrees. Therefore, the total period studied (249 days) is longer than the average breeding period. We collected a total of 4,518 bird-days of continuous wet/dry data during the breeding period. We did not monitor the tagged birds during the periods of deployment and so we have no information on their breeding status except at the time of deployment.

**Figure 1:**
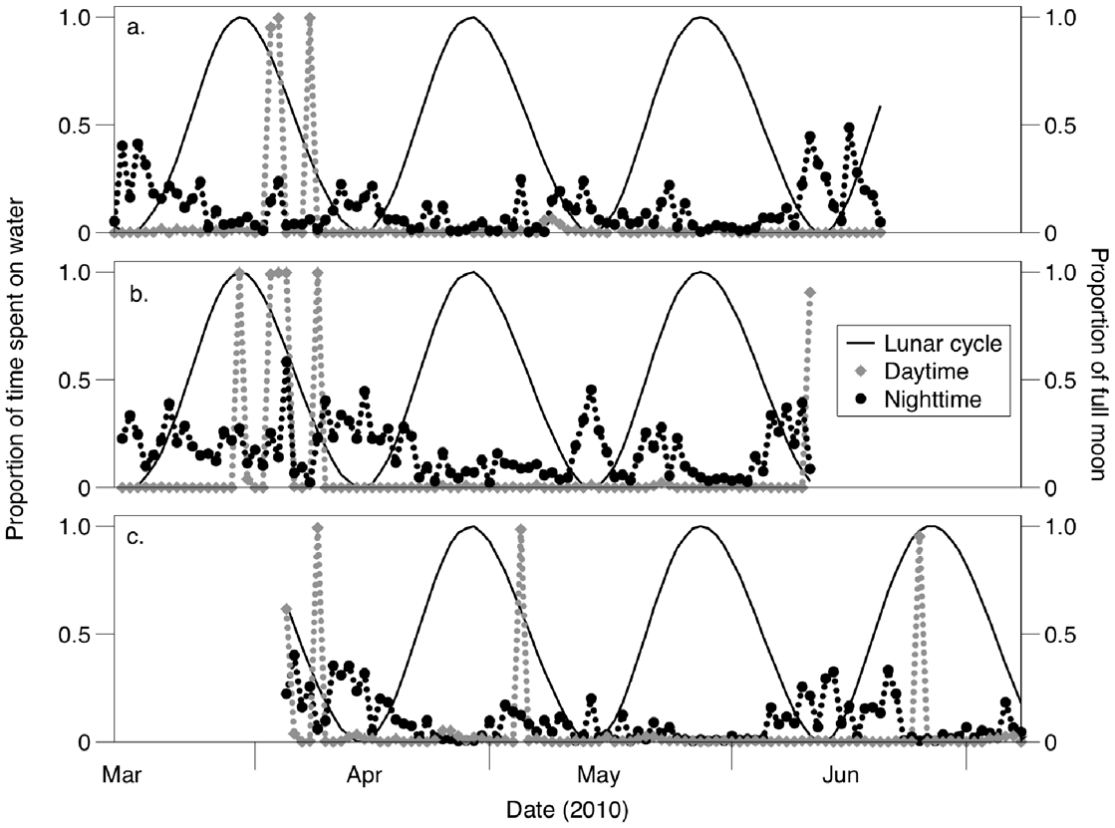
Percentage of time spent on water of three swallow-tailed gulls (*Creagrus furcatus*) during the breeding season. Black circles represent nighttime values and grey circles daytime values; solid black line represents the lunar cycle.

The activity patterns of three birds across their breeding attempts are shown in Figure 1. Throughout the breeding season, daytime values of percentage of time spent on water remained very low and near zero, with the exception of eleven occasions; we deduce that birds did not return to their nests on these occasions, staying on the sea surface to rest throughout the daylight hours. The proportion of time spent on water at night varied with lunar phase for these birds as well; most noticeably, the proportion was close to zero during full moons and increased up to 49% during new moon periods.

**Figure 2:**
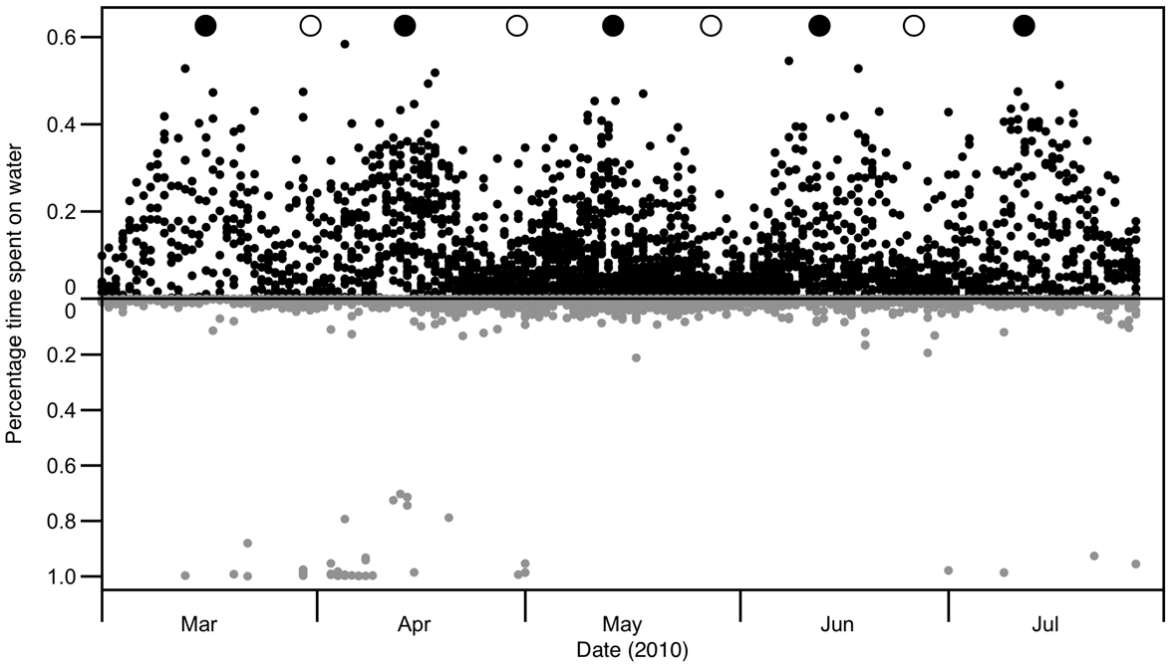
Percentage of time spent on the water by day (grey) and night (black) for 37 swallow-tailed gulls (*Creagrus furcatus*) during the breeding period on Española Island. Upper filled and unfilled circles represent new and full moons, respectively.

**Figure 3:**
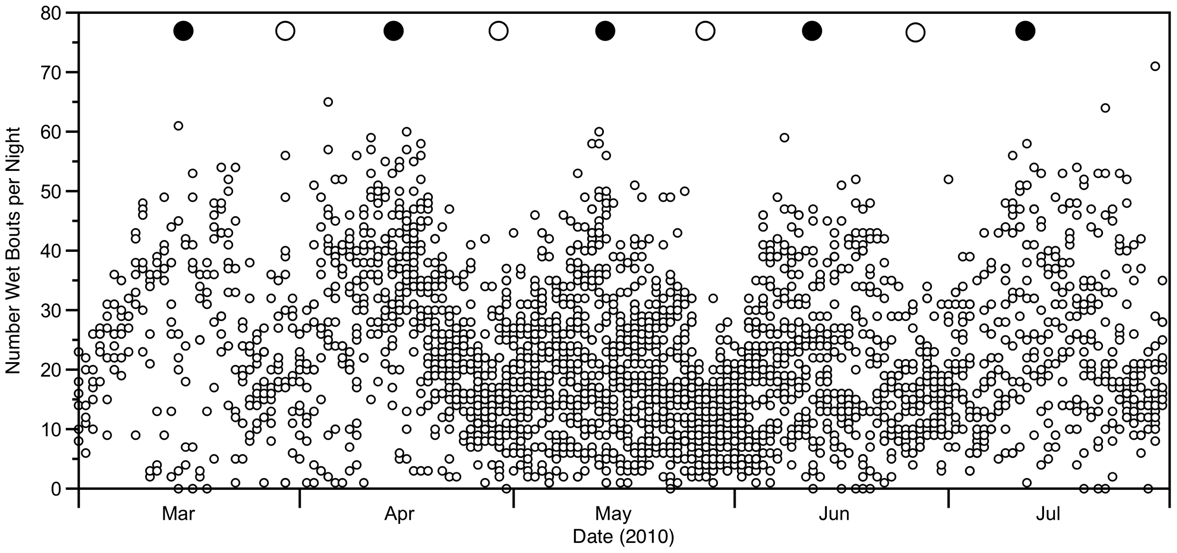
Number of landings on the surface of the water at night for 37 swallow-tailed gulls (*Creagrus furcatus*) during the breeding period on Española Island. Filled and unfilled symbols at top represent new and full moons, respectively.

**Figure 4:**
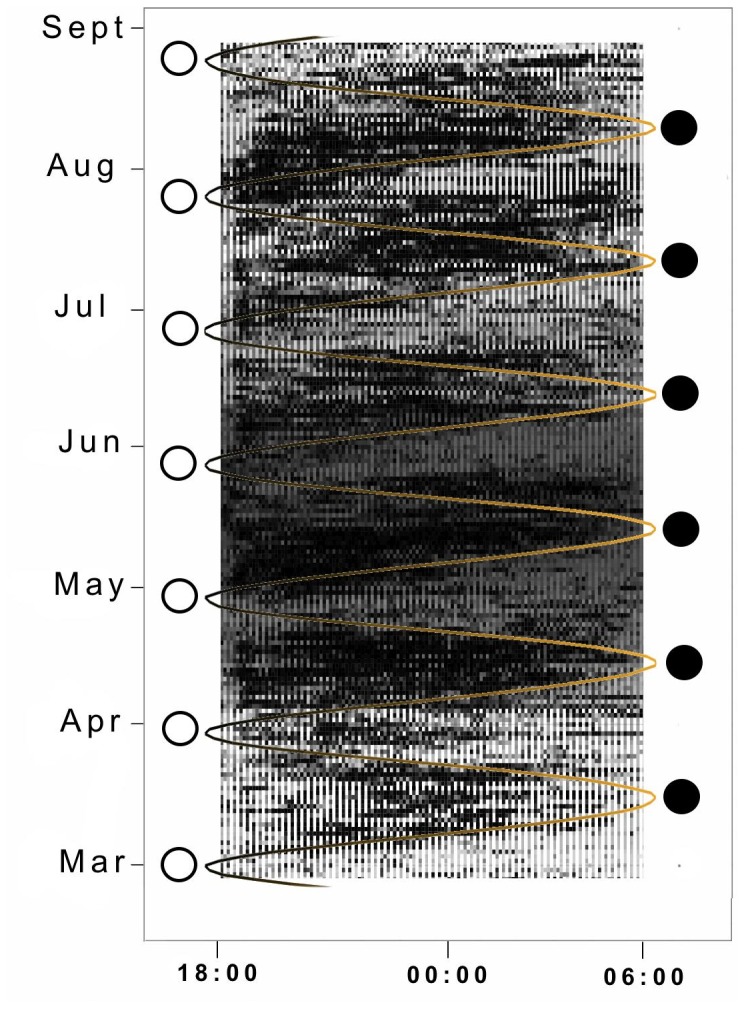
The nighttime activity patterns of 37 swallow-tailed gulls (*Creagrus furcatus*) during the breeding period from Española Island, during the study period extending from March to September 2010. Each small square represents the mean proportion of time the sensors were wet during 10-minute blocks throughout each night of the study. Darker blocks indicate higher proportions of time wet and lighter blocks indicate small proportions of time wet. Curved line and circles represent the lunar cycle from full (black line, open circles) to new moon (yellow line, filled circles).

The proportion of time at night that breeding birds spent on the water (at-sea activity) followed a rhythmic pattern that coincided with the lunar cycle (Fig. 2). Likewise, the number of nocturnal wet bouts varied with lunar phase, peaking during new moons and falling during full moons (Fig. 3). The proportion of time spent on water, our response variable, was strongly correlated with the number of wet bouts (R^2^ = 0.70, P<0.0001, slope = 97.5). The wet activity was clearly reduced during the brightest period of the cycle, the full moon, as shown by the clear, almost horizontal bands in Figure 4. Bands of wet activity (dark) and dry periods (clear) are not perfectly horizontal, related to the daily shift of the time of moonrise and moonset that occurs during the lunar cycle.

### Model Results

**Figure 5:**
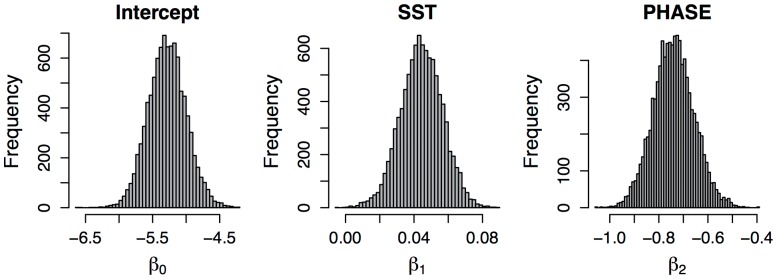
Histograms of MCMC samples depicting the marginal posterior distributions for each of the population-level coefficients.

All population-level coefficients were significant (no credible intervals overlapped zero; Fig. 5, Table 1). The results of our modeling efforts indicate a positive relationship between sea surface temperature and the probability of birds being wet in the population as a whole, and a negative relationship between the probability of a bird being wet and increased illumination related to the moon phase.

**Table 1:** Population level posterior means, standard deviations, and 95% credible intervals from a fit of the hierarchical model.

Parameter	Posterior Mean	Posterior SD	Posterior 95% CI
*Intercept*	–5.82	0.283	(−5.83, −4.71)
*SST*	0.04	0.012	(0.02, 0.07)
*Phase*	−0.74	0.083	(−0.90, −0.57)

That is, these results pertain to the estimation of population coefficients 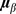, rather than the individual coefficients 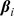.

**Figure 6:**
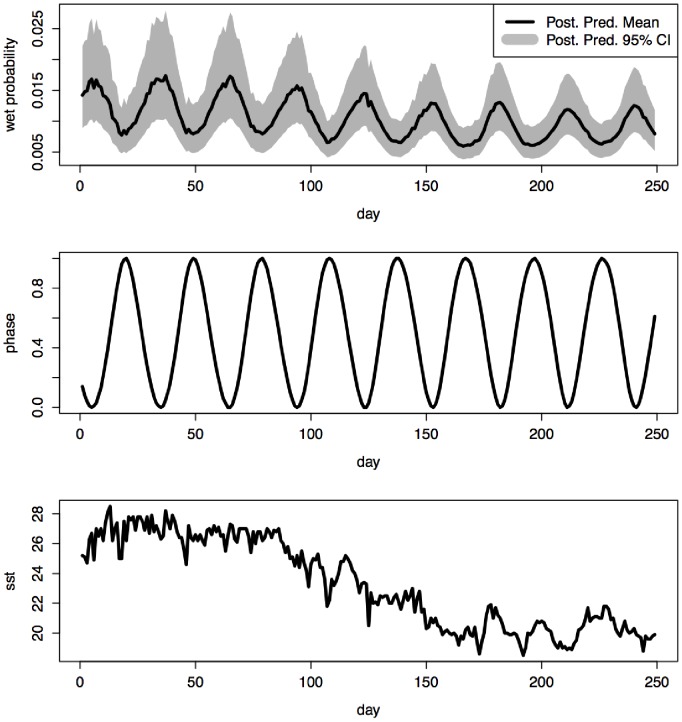
Posterior predictive distribution for *p* (probability of wet) for each of the nights of data collection. The grey area represents the posterior predictive 95% credible interval for this quantity while the solid line represents the posterior predictive mean. Bottom two panels represent moon phase and SST, respectively. Numbers above represent the month of the year, vertical grey lines separate each month accordingly.

The posterior predictive distribution for the probability of wet (i.e.,) was obtained by sampling the full-conditional distribution of within the MCMC algorithm for each day on which data were collected. Figure 6 shows a periodic effect of moon phase on the wet probability, reaching its maximum during full moons, coupled with a much larger scale periodicity, appearing as a downward trend linked to decreasing sea surface temperatures over the period of the study.

## Discussion

Our data show a clear negative association between the at-sea behavior of breeding swallow-tailed gulls and the lunar cycle. The number of wet bouts increased during new moon periods and the percentage of time on water was highest during the darkest periods of each month. Consequently, our results support the hypothesis that swallow-tailed gulls increase their foraging activity when prey is most available; that is, the gull’s presence on the water coincides with the availability of prey following the DVM cycle [8]. Other variables, such as the forager’s breeding stage (which we were not able to include in our model), may also explain some variation in foraging behavior, but the strong signal that we have detected from lunar cycle indicates its predominance.

Tropical waters, in general, have different food web structures, are less productive, less structured, and less predictable than are waters of temperate and polar regions [37]. It has been proposed that selection has favored different foraging strategies in seabirds from temperate or polar compared to tropical waters [38]. For example, Ballance et al. [15] suggested that a good strategy for locating prey, for pelagic birds in the tropics, would be simply to look for them at night due to increased prey availability at the sea surface. Swallow-tailed gulls appear to do just that, and have adaptive characteristics such as large, night-adapted eyes and no discernible melatonin rhythm, to exploit nocturnal conditions at-sea [39]. Therefore, it is not surprising that they have become attuned to the fluctuation in prey availability due to moonlight changes throughout the lunar cycle, consistent with other studies showing that birds adjust their daily patterns of foraging behavior to match activity patterns of their prey [17], [40], [41].

Swallow-tailed gulls, like other seabirds, match their nocturnal activity patterns to the lunar cycle. Swallow-tailed gulls have specialized to forage during nighttime, and they become more active during the darker periods of the month, with peak activity during the new moon. In this respect, they resemble the nocturnal Galápagos fur seal, rather than other seabirds. Changes in the foraging patterns of fur seals over the lunar cycle correlate with the suppression of the vertical migration of prey by lunar light, and consequently, the fur seal’s feeding efficiency might be much higher on dark nights [42], [43]. Likewise, the activity patterns of *Lophostoma silvicolum* bats decreases significantly during the brightest nights of the month, and the reduction in activity is strongly correlated with the behavior of prey in connection with the lunar cycle [5]. Frigatebirds (*Fregata* spp.) pursue and kleptoparasitze swallow-tailed gulls during daylight hours [26], but not at night, at least in the vicinity of the breeding colony (pers. obs.). Kleptoparasitism by frigatebirds could have contributed to the evolution of nocturnality [25], although this remains to be thoroughly tested, but probably not to the pattern revealed by this study.

Swallow-tailed gulls capture their prey by surface plunging, and have access only to the upper 1 m of the water column (S. Cruz, unpublished data). Therefore, changes in the depth of their prey are especially significant because vertically migrating fish or squid are out of reach to gulls when at depths greater than 1 m [29]. During well-lit nights, such as full moon periods, it is possible that the foraging efficiency of gulls is compromised. This notion seems to be supported by our data: the proportion of time spent in water at night of individual birds during full moon is very low (∼zero). We suggest that birds either stay on land attending their nest or chick or encounter less prey at sea during well-lit nights, which results in fewer attempts to capture them and therefore less time in the water overall. This pattern is evident both at the individual and population levels.

The SST around the Galápagos Islands had a discernible positive relationship with at-sea behavior of swallow-tailed gulls. Overall, foraging activity decreased with lower SST. We suggest two possible hypotheses that could explain this observation: (1) increased productivity due to colder waters around the Galápagos means that prey are more available when the water is colder, swallow-tailed gulls capture more prey per landing, and we observed this as birds spending less time on water; (2) colder SST may have reduced the availability of swallow-tailed gull prey, due to their temperature preferences, resulting in poorer foraging conditions, fewer prey captures and, therefore, less time spent on the water. We are unable to test these hypotheses at present because data on prey availability do not exist.

The Bayesian hierarchical model used in this study allowed us to establish the link between lunar cycles and at-sea activity patterns of swallow-tailed gulls and provided intuitive and meaningful inference. Furthermore, a large sample size both in number of individuals and days recorded provided a robust dataset from which we derived our conclusions. Moreover, our approach offers an alternative method for modeling information from activity loggers and environmental data, which could be useful for the increasing number of tracking studies of seabirds around the world. We provide a specific example of how animals can adjust behaviorally to environmental changes. Our study demonstrates how animals can use strong and predictable environmental cues, such as the lunar cycle, to inform behavioral decisions [44], [45]. Finally, we recommend that efforts be increased to study tropical species that show contrasting ecological traits from those in temperate regions, so that management and conservation strategies in the tropics are informed by the best available and relevant data rather than less applicable temperate zone information.
